# A serpiginous lesion of scrotum

**DOI:** 10.1186/s13052-022-01224-7

**Published:** 2022-03-03

**Authors:** Sara Romano, Roberto Dall’Amico, Valentina Declich, Egidio Barbi

**Affiliations:** 1 Dipartimento Di Scienze Mediche e Chirurgiche e Della Salute, Università Degli Studi Di Trieste, Trieste, Italy; 2 S. Maria Degli Angeli Hospital - Paediatric Department, Pordenone, Italy; 3 Institute for Maternal and Child Health - IRCCS “Burlo Garofolo” - Paediatric Department, Trieste, Italy

**Keywords:** Child, Scrotum, Scrotal raphe cyst, Infection, Serratia marcescens

## Abstract

**Background:**

Median raphe cyst is usually benign and asymptomatic male genitalia lesions. Although uncommon, infection may be a complication.

**Case presentation:**

We report the case of a 4-year-old child presented to the emergency department for a serpiginous and redness lesion extended from the basis of the penis until the perineum. An infected median raphe cyst was suspected, and the patient underwent surgical treatment and antibiotic therapy with complete resolution of symptoms. Liquid culture resulted positive for *Serratia Marcescens*.

**Conclusion:**

Infection is a rare complication, especially in childhood. To prevent relapses and clinical symptoms, the majority of authors recommend surgical excision followed by primary closure. In case of infections caused by *Serratia Marcescens*, chronic granulomatous disease should always be rule out.

## Background

Median raphe cysts are an embryologic fusion abnormality of the male genitalia that develop along the median penile raphe from the meatus to the perineum [1]. Their clinical presentation is usually in the form of a watery solitary cyst, multiple cysts, or canal-like lesions in the ventral midline part of the penis and perineum [2]. Their diagnosis in childhood is rare, probably because up to 75% of cases are asymptomatic [2–4]. Therefore, patients often present during second to third decades of life [5]. Infection, rupture, and sexual interference are the most common complications [4].

We present the case of a child with an infected median raphe cyst who underwent surgical and antibiotic treatment with complete resolution of symptoms. Infection is a rare complication, especially in children. This is the first case described in literature in which the culture isolated a microorganism more commonly responsible of infections in patients affected by immunodeficiencies.

## Case presentation

**Fig. 1:**
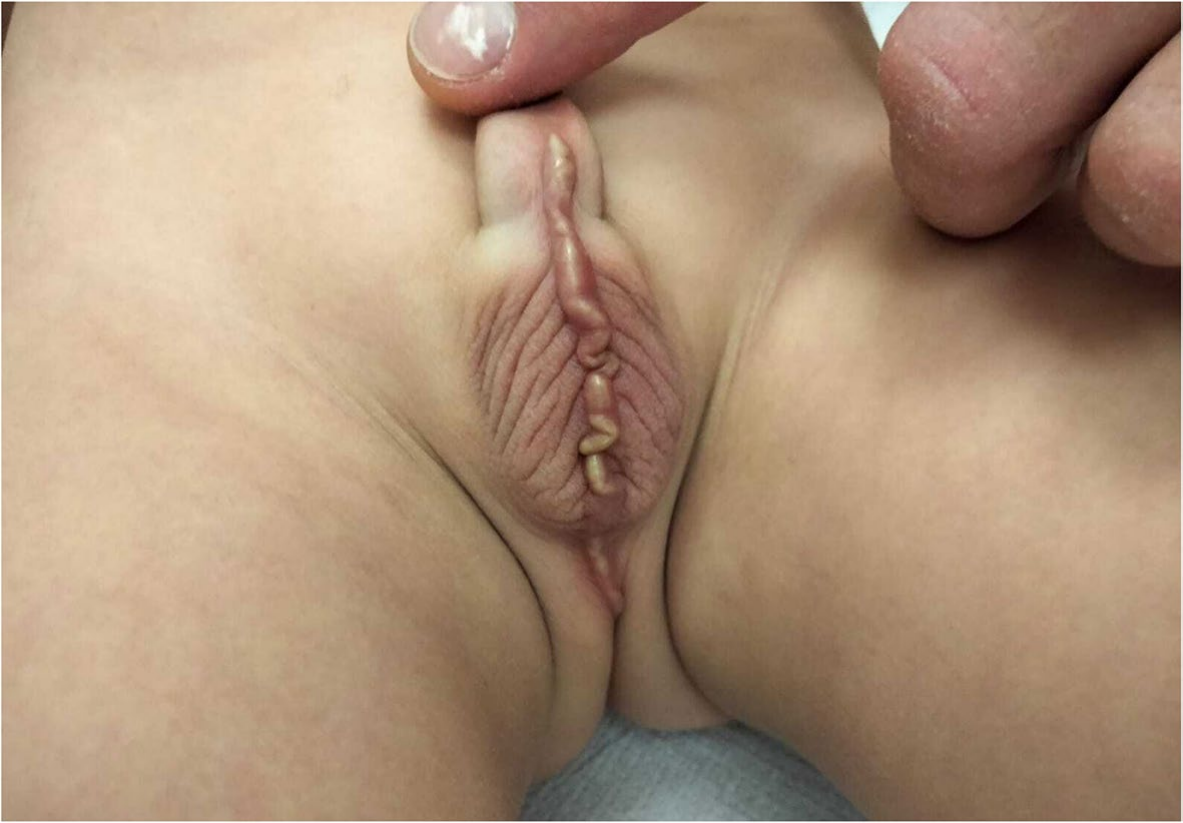
Serpiginous lesion extended for 4 cm from the basis of the penis all along the scrotum in the midline

**Fig. 2:**
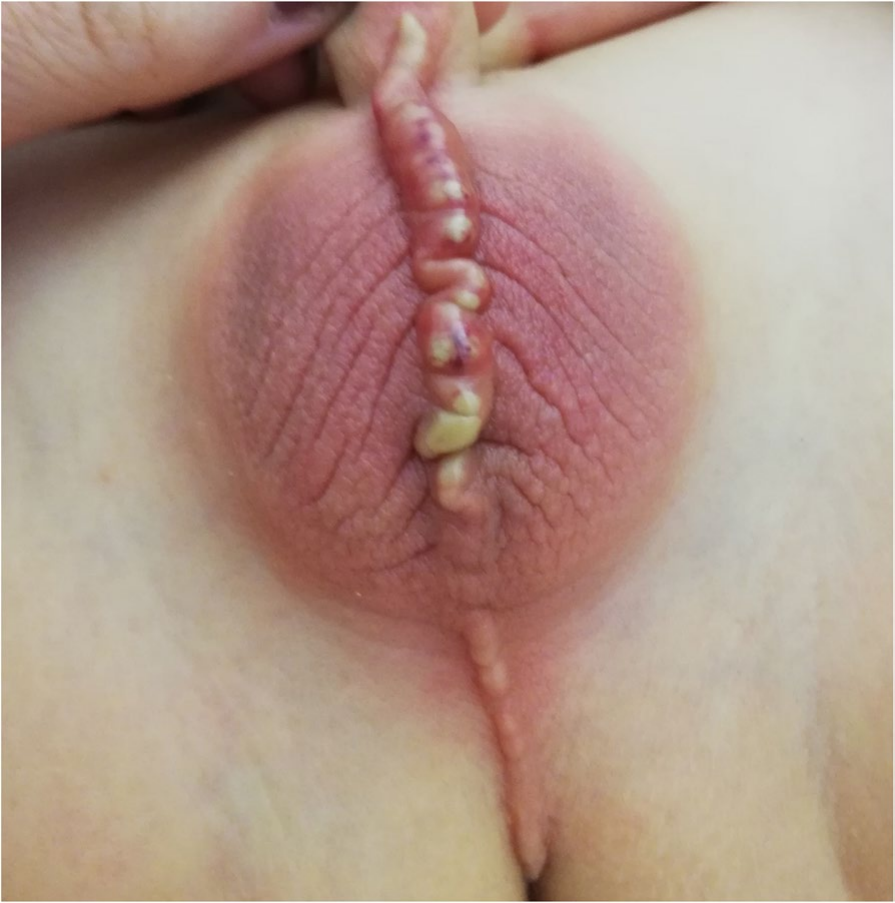
Scrotum cellulitis with pus secretion from the lesion

A 4-year-old-boy presented to the emergency department for a four-day history of discomfort during peeing. The urine analysis was normal. No fever was detected. Past medical and family history were no significant. The clinical examination of genitals showed a serpiginous and redness lesion extended from the basis of the penis and scrotum all along the midline until the perineum (Fig. 1). It was mostly tender and painful to palpation. An empirical broad spectrum antibiotic therapy with oral amoxicillin/clavulanic acid at a dosage of 80 mg/kg and topical tobramycin was started. The day after the child complained of severe pain of the scrotal region with worsened redness and swelling and pus discharge (Fig. 2). An ultrasound scan showed thickening and hypo echogenicity of the scrotal raphe. The abdomen ultrasound was normal, no genitourinary anomalies were detected.

Based on the clinical examination, the diagnosis of canal-like median raphe cyst was suspected. In this case the cyst was complicated by infection. Considered the poor respond to antibiotic therapy and the symptoms worsening, a surgical treatment was decided, and the lesion was incised to drain the purulent liquid. The wound was medicated with chlorhexidine and oral antibiotic therapy with amoxicillin/clavulanic acid was continued for 10 days. *Serratia Marcescens* was isolated from the cultures, but unfortunately the material was not considered sufficient for histopathological confirmation. Symptoms completely resolved after treatment. The patient underwent to surgery follow up and no other interventions required. However, *Serratia Marcescens* is one of the most common pathogens responsible of infection in children with chronic granulomatous disease (CGD), especially in the genitourinary system. Therefore, the assessment of nicotinamide adenine dinucleotide phosphate (NADPH) oxidase function was performed. The complex’s activity resulted intact excluding the immunodeficiency.

## Discussion

Median raphe cysts are congenital uncommon lesions of the male genitalia developing along the median penile raphe from the meatus to the perineum [1]. They result from “tissue trappings” during the intra-uterine process of closure of urethral or genital folds [4]. Their clinical presentation is usually as a flesh to yellow solitary cyst, multiple cysts, or canal-like lesions in the ventral midline part of the penis and perineum [2]. The penile shaft is the most frequent location, followed by subcoronal, scrotal and perineal areas [1]. Cysts may sightly fluctuate in size throughout life [3]. Three histologic patterns have been described: 1) urethral type lined with pseudostratified columnar epithelium, such as the urothelium; (2) epidermoid type, with squamous stratified epithelium; and (3) mixed type, with both epitheliae. The urethral type is the most frequent (70.1%) [4]. Their diagnosis in children is probably underreported with a median age at diagnosis of 24,6 years [5]. In fact, as cysts do not connect with urethra, up to 75% of cases are asymptomatic and infection is a rare complication [2, 6]. Other described complications are the cysts rupture and sexual interference in adulthood^4.^ To prevent relapses and clinical symptoms, the majority of authors recommend surgical excision followed by primary closure^1,5^.

In case of infection, the most common organisms found in adults’ patient are *Neisseria gonorrhoeae, Trichomonas vaginalis* and *Staphylococcus aureus *[6]. This case is unique in literature because the drainage liquid culture resulted positive for *Serratia Marcescens*, a common pathogen causing infections in patients affected by immunodeficiencies. In particular, chronic granulomatous disease (CGD) characterized by the defect in superoxide production, is the first immunodeficiency to rule out. Patients with CGD have an increased susceptibility to a narrow spectrum of pathogens including, beyond *Serratia Marcescens*, *Staphylococcus aureus*, *Burkholderia cepacia* complex, *Nocardia* species, *Aspergillus* species, *Salmonella* species and *Mycobacterium tuberculosis*. Most patient initially present within 5-years-old with lymphadenitis, abscesses especially of the liver and perianal region, pulmonary infections, osteomyelitis, and sepsis. Genitourinary and gastrointestinal systems are generally affected by granulomatous inflammation. The gold standard for diagnosing CGD is flowcytometric dihydrorhodamine (DHR) neutrophil respiratory burst assay to assess the nicotinamide adenine dinucleotide phosphate (NADPH) oxidase function [7, 8].

## Conclusion

The diagnosis of median raphe cysts is clinical. The condition is more often asymptomatic in childhood, but infection or cyst rupture could occasionally occur. In these circumstances, the majority of authors recommend surgical excision.

Our case is the first described in literature in which cyst’ liquid culture resulted positive for *Serratia Marcescens.* Every infection caused by this gram-negative organism should raise the suspicion of an underlining immunological disorder, and chronic granulomatous disease should always be excluded.

## Data Availability

Data sharing is not applicable to this article as no datasets were generated or analysed during the current study.

## Abbreviations

CGD: Chronic granulomatous disease
DHR: Dihydrorhodamine
NADPH: Nicotinamide adenine dinucleotide phosphate
